# A pilot course on climate and health for students and faculty in the health professions

**DOI:** 10.3389/fpubh.2026.1714680

**Published:** 2026-04-07

**Authors:** Trisha Dalapati, Jennifer M. Lawson, John H. Lohnes, Allyson S. Sutkowi-Hemstreet, Perri A. Morgan, Denise M. Nepveux, Valerie K. Sabol, Andrew J. Muzyk, Brian G. McAdoo, AnnMarie L. Walton

**Affiliations:** 1 Duke University School of Medicine, Durham, NC, United States; 2 Department of Pediatrics, Duke University School of Medicine, Durham, NC, United States; 3 Division of Physician Assistant Studies, Department of Family Medicine and Community Health, Duke University School of Medicine, Durham, NC, United States; 4 Division of Physical Therapy and Rehabilitation Science, Department of Family Medicine and Community Health, University of Minnesota, Minneapolis, MN, United States; 5 Occupational Therapy Doctorate Division, Department of Orthopaedic Surgery, Duke University School of Medicine, Durham, NC, United States; 6 Duke University School of Nursing, Durham, NC, United States; 7 Practice of Medical Education, Duke University School of Medicine, Durham, NC, United States; 8 Division of Earth and Climate Sciences, Duke University Nicholas School of the Environment, Durham, NC, United States

**Keywords:** climate change, health professions, healthcare systems, interprofessional education (IPE), sustainability

## Abstract

**Purpose:**

Managing the health impacts of climate change will require the coordinated expertise of all health professions. Therefore, trainees and practitioners need to learn about climate change and its health impacts in an interprofessional setting. Accordingly, faculty members and students representing different health and climate sciences at Duke University created an interprofessional pilot course for students and faculty in the health professions.

**Methods:**

In addition to teaching fundamental concepts regarding the science of climate change and downstream health consequences, a solutions-based approach was utilized to highlight how individuals and healthcare systems can address climate change. The course format included asynchronous virtual modules and in-person service-learning opportunities to engage learners directly in community-based climate mitigation and adaptation efforts.

**Results:**

A validated pre- and post-course survey called CHANT assessed health professional learners’ awareness, motivations, and behaviors related to climate change and health. Learners were already aware of climate change, but only around 60% reported being at least somewhat familiar with the contribution of the U.S. healthcare system to greenhouse gas emissions. Although over 90% of learners already embraced climate-protective behaviors and conversations at home, almost 40% of learners reported never or rarely engaging in these same behaviors and discussions in the workplace. Learners provided overall positive feedback regarding satisfaction with course load, pace, and resources. Students reported interest in more in-person activities, climate justice and advocacy opportunities, and information on healthcare sustainability and preparedness.

**Conclusion:**

In response to the feedback received from learners, an online course has been developed using the pilot course materials to increase accessibility to all learners in healthcare, including practicing healthcare workers and administrators from diverse professional disciplines.

## Introduction

The climate crisis is a health crisis. According to the World Health Organization (WHO), “Climate change is the single biggest health threat facing humanity” (1). The health impacts of climate change are far reaching, impacting most human physiologic systems. Higher temperatures and extreme weather events such as hurricanes, floods, and wildfires may lead to health consequences including asthma exacerbations, myocardial infarction, renal failure, heat stroke, vector borne illnesses, malnutrition, developmental concerns, violence, anxiety, displacement and forced migration (2). Although climate change amplifies underlying health risks for all, the climate crisis disproportionately affects vulnerable populations including children, pregnant people, older adults, individuals with disabilities and chronic illnesses, and historically under-resourced communities (3).

To address the relationship between climate change and health, it is critical to recognize the impact of health systems on climate change. Globally, the healthcare industry accounts for 4–5% of greenhouse gas (GHG) emissions (4, 5). The U.S. healthcare system contributes to 8.5–10% of the country’s GHG emissions (4, 5). The healthcare community must first recognize that these emissions exacerbate climate change and thus endanger its mission to protect and promote wellbeing. As stewards of health, the healthcare industry must move to mitigate the impacts of climate change and create solutions for preparedness and resilience.

Despite the known inextricable relationship between climate change and health, most healthcare professionals have limited knowledge and formal education in this area. To provide high-quality care, they must recognize the impact of climate change on health and learn to provide climate-informed care for their patients. In addition, it is important for healthcare professionals to be cognizant of the operational impacts of health systems on climate change and their professional roles and responsibilities in this context. Climate and health education for healthcare professionals is not only essential for high-quality direct patient care but for responsible stewardship of the surrounding environment. This education is critical not only for clinicians but also for researchers, educators, non-clinical staff, and administrators. While many U.S. medical schools are integrating climate change and health into their curricula, rising from 25 to 52% over recent years (6), significant knowledge gaps remain among trainees, faculty, and practicing professionals across all healthcare professions. Climate change education is particularly lacking in the interprofessional healthcare arena (7, 8), where there is promise for development of clinically meaningful, practical solutions.

To address the need for interprofessional education about climate change and health at our institution, we, an interprofessional team of faculty members and students at Duke University, hereon referred to as a Community of Practice (CoP), designed and piloted “Climate Change and Health for Healthcare Professionals,” an asynchronous, hybrid course with virtual modules and in-person community service-learning opportunities. We intentionally recruited both student and faculty learners from across health professions programs and schools. We assessed climate change knowledge, motivations, and behaviors before and following participation in the course. Importantly, we sought to highlight climate change as a social driver of health and to deliver our content through a solutions-oriented perspective which is a future-oriented, goal-directed approach to solving problems with the people affected by the problem.

## Methods

This study was deemed exempt from continued review by the Duke University Health System Institutional Review Board.

### Needs assessment

In January 2023, a CoP from a variety of professional backgrounds and disciplines at Duke University (Nicholas School of the Environment, School of Nursing, School of Medicine), convened to discuss and identify the state of interprofessional education about climate change and health at our institution and beyond. The CoP identified four goals for a course designed for students and faculty: (1) increase climate and sustainability knowledge, skills, and attitudes by teaching the causes, context, and health consequences of climate change; (2) emphasize the importance of interprofessional education to create clinically meaningful, solutions-oriented impact; (3) organize community-based service-learning events to catalyze direct and sustained engagement with climate-protective activities, and (4) familiarize participants with the Duke University Hospital System infrastructure to illustrate the contribution and impact of healthcare systems on the environment. Importantly, these goals aligned with the Duke University’s Climate Commitment, a university-wide initiative launched in 2022 that aims to engage all Duke community members in creating solutions countering the impacts of climate change through education and research.

### Course development and implementation

To promote the interdisciplinary aspect of climate change and health education, we prioritized creating a learning format that would allow participation of learners from multiple programs and disciplines and with different schedules. We used the Planetary Health Education Framework to guide our selection of the content for the course (9) and began the course by explaining how human activity has been the dominant influence on climate and the environment at both local and international levels. We recognized the centering of interconnection within nature and ensured inclusion of related videos, reflection exercises and other modalities throughout all the content areas. Our solutions-oriented approaches included content centered on equity and social justice, systems thinking and complexity (especially focused on health systems sustainability), and movement-building and systems change, all of which included engagement with impacted individuals and communities (Supplementary Table 1 for module descriptions).

We grounded instructional design decisions in Malcolm Knowles’ Adult Learning Theory (10, 11). The six principles of andragogy, the art or science of teaching adults, assume several factors about adult learners. First, adults are intrinsically motivated and ready to learn when they see a need in their lives that learning can help fulfill; our learners self-selected into this course. We assumed the learners were ready and motivated to engage in this content; this freed the instructors to focus on more advanced content and the science of solutions rather than on providing proof of climate change. Adult learners also bring their past education, employment, and life experiences to their learning and often prefer to learn in a social context; as such, we designed optional facilitated discussion sessions hosted by faculty from at least two disciplines, in-person activities, and online discussion forums so learners could share personal and professional stories of engagement. Adult learners tend to have independent learning skills, and these learners came from a variety of disciplines with quite varied schedules. Accordingly, we designed the modules to be asynchronous and self-paced. The final project encouraged our adult learners to generate a novel product that reflected their growth and learning across the course. This final project’s design capitalized on the idea that adults are motivated to learn through problem-centered materials and assignments that are relevant to real life. We accepted a wide range of products as there was no grade assigned to the course. We also saw the wide range of products as an opportunity to gather information on what the learners would most naturally choose.

We chose an asynchronous format, which would include (1) recorded lectures and materials posted on an online learning platform with the opportunity for discussion posts and timely feedback and comments from CoP members and peers, and (2) frequent in-person activities to enable relationship-building among learners and to highlight local community challenges and solutions concerning climate change mitigation and adaptation.

Throughout the course development and implementation (May 2023 to April 2024), the CoP met weekly for one hour to plan and review the course goals and materials. Course materials were devised through the summer of 2023 with the goal of launching the course in October 2023 and completing the course in April 2024. Seven modules were mapped, and each module was co-developed by two different CoP members (Supplementary Table 1). Module content included prerecorded lectures, recommended but optional readings and videos, discussion questions to answer on the online platform, and in-person activities. Modules were vetted and revised by the CoP. Beginning in October, a new asynchronous module was launched each month. We created and distributed a weekly email newsletter to highlight local and national climate news, opportunities, and resources for further learner engagement. Additionally, virtual optional facilitated discussion sessions for each module were offered by CoP members.

We recruited learners starting in August 2023 via emails sent to five Duke University health professional programs and schools [Doctor of Medicine (MD), School of Nursing (SoN), Occupational Therapy Doctorate (OTD), Physician Assistant (PA), Doctor of Physical Therapy (DPT), and the Nicholas School of the Environment (NSOE)]. The same recruitment email was sent to the Campbell University School of Pharmacy in North Carolina. The recruitment email included a short survey asking applicants to identify their affiliated program and respond to selected questions from the Climate and Health Tool (CHANT) (12) that evaluated knowledge, motivation, and behaviors regarding climate change and health.

### Tools to evaluate learning and course format and materials

CHANT is a freely available, validated online survey that takes approximately 10 minutes to complete and asks respondents about awareness, motivation, and behaviors related to climate change and health. The items were developed with the input of content experts in 2017, and the tool was psychometrically analyzed and then utilized in several studies (12–14). It was initially utilized with nurses (13) and expanded to all health professionals in 2020. CHANT has been used in over 20 countries and has been translated into ten languages. As the survey responses are anonymous, we contacted the survey creators to request a unique link that was distributed and used only among our pilot course experience learners. The same unique link was used for the pre- and post-course assessment. Learners enrolled in the course were asked to complete the CHANT within 1 week of enrollment in the course. As part of the final module, learners were asked to complete the same survey to assess if and how the pilot course was impactful. Results were analyzed in aggregate to assess pre- and post-course changes.

In addition to CHANT, we created a course evaluation survey to understand how learners perceived the course load, pace, resources, alignment of the materials with objectives of the course, and online learning platform. Learners had the option to submit narrative comments related to each domain in the survey.

For the final module, learners were asked to create a final project reflecting on their learning throughout the pilot course experience. This project could take the form of an informational flyer or brochure for dissemination to the local community, an opinion or commentary for a lay audience, an essay for a scientific journal, or an art or literary piece. The purpose was to give students an opportunity for creative expression and to apply their learning to a local or scientific community purpose.

### Data visualization and statistical analysis

Data were collated and analyzed using Excel (Version 16.85) and R Statistical Software (v4.3.0) (15). R packages “*likert*” and “*dplyr*” were used to create Figures. Comparisons of pre- and post-course CHANT results were analyzed using the Wilcoxon rank sum test with continuity correction. *p*-values are notated as the following: non-significant (ns) > 0.05; * ≤ 0.05; ** ≤ 0.01; *** ≤ 0.001; and **** ≤ 0.0001.

## Results

### Recruitment and enrollment

Our goal was to recruit two student-learners from each identified program/school and four faculty-learners overall. We received a total of 38 applications (36 student-learners and 2 faculty-learners) with representation from all six Duke-affiliated programs and schools contacted (Supplementary Table 2). We received the most applications from the SoN (10) and PA program (9). We received the fewest applications from the NSOE (4) and MD program (3). We did not receive applications from the Campbell School of Pharmacy.

All applicants were invited to enroll in the course. Course enrollment was achieved by enrollment in the online learning platform and completion of the CHANT. Course completion was achieved by completing both the CHANT again and course evaluation survey. Ultimately, 30 learners enrolled in the course, 23 completed the course evaluation, and 21 completed the course (70% completion). The largest number of learners completing the course were in the PA (6) and DPT program (6) and the fewest were in the MD program (2) and NSOE (1) (Supplementary Table 2).

### Engagement in online vs. in-person activities

Using the online platform, we tracked the activity of the 21 learners who completed the course. This ranged from 3 to 34 h with an average of approximately 18 total hours spent engaged with the online content. Learners utilized the discussion forum and submitted thoughtful posts for each module. We offered four virtual optional facilitated discussion sessions for the first four modules during weekday afternoons, and three types of in-person events (Supplementary Table 1): (1) two Climate Fresk sessions on weeknights, (2) two volunteer sessions with REMEDY at Duke, a medical supply recovery program with international partners, on weekend mornings, and (3) a street tree planting session on a Friday afternoon. Learner attendance at these events was generally low, ranging from 2 to 4 learners.

### Increased understanding of the role of healthcare in climate change

**Figure 1:**
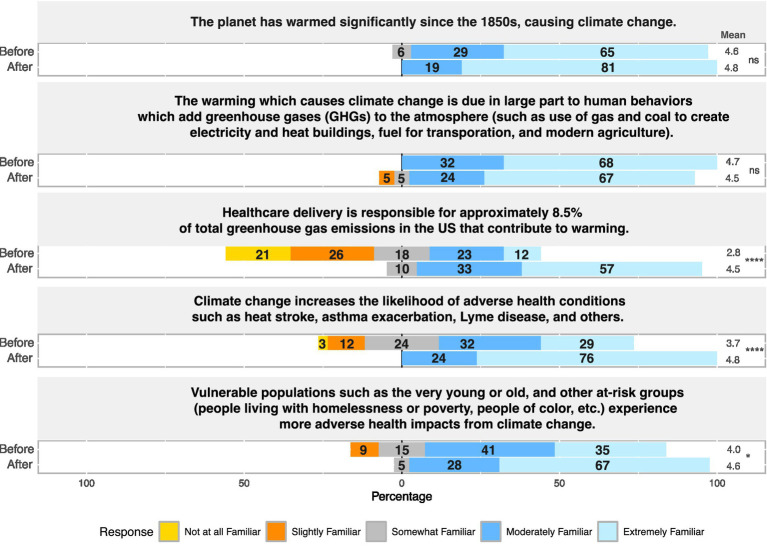
Results summarizing the familiarity of learners with the following evidence-based statements.

Learners completed the CHANT the week prior to the course start date and within a month of finishing the course, which allowed us to measure changes in awareness, motivation, and behaviors related to climate change. The results highlighted that many learners perceived themselves to be already somewhat or extremely familiar with global warming and human behaviors that contribute to greenhouse gas emissions before the course. However, learners reported significantly increased knowledge on key points including the contribution of the healthcare sector to greenhouse gas emissions (*p*-value = 1.13 × 10^−5^), the adverse health effects related to climate change (*p*-value = 2.13 × 10^−4^), and the disproportionate risks affecting certain groups like children, older adults, individuals without housing, and historically under-resourced communities (*p*-value = 1.43 × 10^−2^) (Figure 1). Many learners reported frequently observing respiratory conditions (asthma, allergies, and worsening pulmonary conditions) and mental health issues among patients both before and after the course (Supplementary Figure S1). However, learners reported not frequently observing vector-borne illnesses, extreme heat illness, or physical trauma related to disasters before or after the course (Supplementary Figure S1).

**Figure 2:**
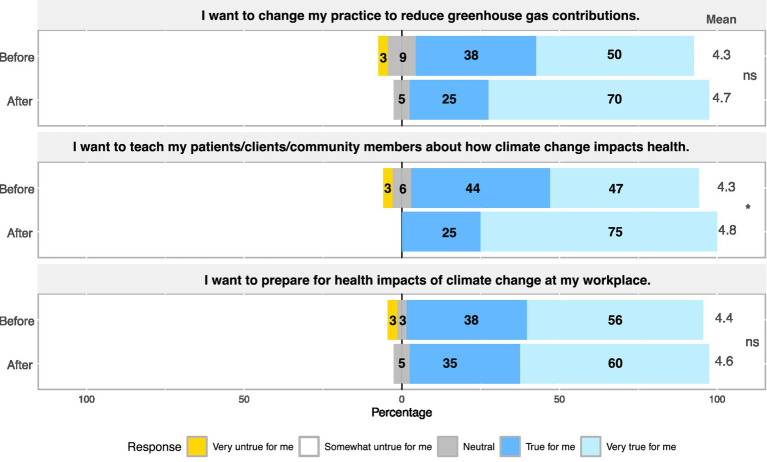
Results summarizing desires and motivations of learners related to climate-protective behaviors.

**Figure 3:**
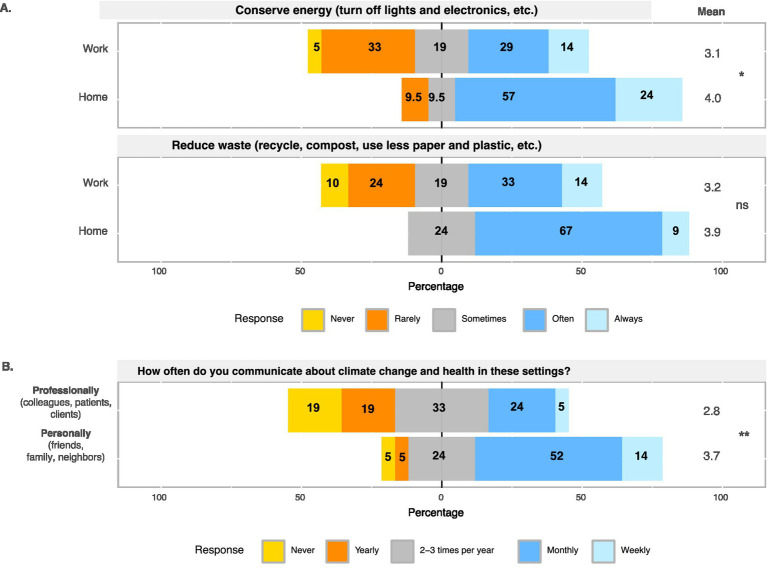
Results summarizing learners’ climate-protective behaviors **(A)** and communication about climate change **(B)** in professional and personal settings after course completion.

Before the course, most learners felt strongly that they wanted to reduce their GHG contributions, teach others about how climate change impacts health, and prepare for the impacts of climate change in the professional setting. Importantly, there was a statistically significant increase in the desire to teach patients, clients, and community members about the health impacts of climate change following participation in the pilot course (*p*-value = 3.61 × 10^−2^; Figure 2). Most learners indicated that they were already practicing climate-protective behaviors at home before the course, including conserving energy, limiting use of gasoline, reducing waste, and consuming foods that require less resources; these types of behaviors generally increased following participation in the pilot course (Supplementary Figure S2). However, despite their desire to prepare for the consequences of climate change at work and to inform patients and clients, learners still reported practicing significantly less frequent climate-protective behaviors at work compared to at home such as conserving energy after the course (*p*-value = 2.35 × 10^−2^; Figure 3A; Supplementary Figure S3). Learners were also significantly more likely to discuss climate and its health impacts in their personal lives compared to in professional settings (*p*-value = 9.69 × 10^−3^; Figure 3B).

Importantly, the CHANT results highlighted that most students were already concerned about the diverse impacts of climate change and remained so after the course. Additionally, learners expressed limited optimism that humans will adequately prepare for the impacts of climate change or prevent further climate change.

### Course evaluation and areas for improvement

Results from the course evaluation survey highlighted the strengths and areas of improvement of the course format and content (Table 1). Although there was limited participation in the in-person activities offered due to schedule conflicts and the optional nature, several learners expressed a desire for more in-person interactions, especially service-based and community activities. Learners who did attend in-person activities found them stimulating and valuable to increase their understanding of online course material. The interprofessional nature of the course was widely appreciated. Interestingly, although most learners were enthusiastic about the asynchronous format and flexibility, some learners suggested creating more specific deadlines for reviewing content and submitting assignments to ensure accountability in a voluntary course.

**Table 1:** Aggregate course evaluation results.

Topic	Response	Response % (number)
Course load	Very light	4 (1)
	Light	3 (7)
	Moderate	61 (14)
	Heavy	4 (1)
	Very heavy	0 (0)
Pace of the course was appropriate	Yes	83 (19)
	No	17 (4)
Satisfaction with resources provided	Extremely satisfied	74 (17)
	Somewhat satisfied	22 (5)
	Neither satisfied nor dissatisfied	4 (1)
	Somewhat dissatisfied	0 (0)
	Extremely dissatisfied	0 (0)
Assignments aligned with objectives and helpful for learning	Yes	91 (21)
	No	4.5 (1)
	No response	4.5 (1)
Technical difficulties encountered	Yes	9 (2)
	No	91 (21)
Level of instructor support	Very good	100 (18)
	Good	22 (5)
	Fair	0 (0)
	Poor	0 (0)
	Very poor	0 (0)
Preferred course format	Asynchronous, mostly online over 2 semesters	Ranked #1
	Asynchronous, mostly online over 1 semester	Ranked #2

In terms of module topics and content, several learners suggested a greater focus on climate justice and on health system sustainability and climate preparedness (Supplementary Table 1). Students voiced an interest in learning the details of the Duke University Hospital System carbon footprint and the solutions underway to mitigate its impacts.

### Final project

Of the 21 students who completed the course, 12 submitted a project. Most submissions were informational flyers (9) focused on topics including extreme heat and safety, outdoor air quality, emergency preparedness, healthcare waste, and local environmental justice efforts. Importantly, all flyers included both background information and actionable steps that could be undertaken at an individual level. The target audience of these flyers was the local Durham community. Two submissions were essays on the topics of air pollution and brain health and were prepared for submission to academic journals. One submission was a poem on the disproportionate impact of climate change on the health of historically underserved communities; the learner submitted it to a local literary magazine.

## Discussion

The response to course recruitment efforts suggested broad but disparate levels of interest in climate change and health education across health professions and environment schools at Duke University. Discrepancies could have been due to factors such as existing courseloads and schedules and prior extracurricular commitments. During the recruitment process, the CoP desired to include pharmacy students and faculty from the Campbell University Doctor of Pharmacy program but did not receive any applications, which may be due to a lack of connection with Duke. Finally, only two faculty members applied for and enrolled in the course. Faculty may require a more targeted recruitment approach, a briefer course commitment, and different incentives for course completion such as continuing education units. A separate learning opportunity may be needed for them in community with one another.

In addition to enrollment, we noted challenges in learner retention as nine learners (30%) did not complete the course. Learners voiced several challenges preventing course engagement including prioritization of credit-bearing coursework beyond this voluntary, non-credit bearing course experience, scheduling constraints, feeling disconnected from the course and peers due to limited in-person activities, and lack of mandatory deadlines. Evaluation comments from learners indicated that the asynchronous format of the course may be essential to increasing enrollment numbers and improving retention as the format allows for flexibility amidst diverse didactic and clinical schedules. However, learners also emphasized a need to connect with other learners in-person.

Results of course evaluations, including narrative comments on content and format, suggest several challenges. To enhance recruitment and retention across schools and among prospective faculty learners, we need to understand and address specific barriers to recruitment and retention among disparate groups. Another challenge is to understand how to meet needs for flexible, asynchronous learning formats (16) while also facilitating authentic interpersonal, interprofessional and community connection that learners need and desire (17). Importantly, the pilot course was created through the efforts of our interprofessional CoP that included trainees and faculty members who worked asynchronously yet met weekly for a year; similarly, learners echoed the need for flexibility of the course yet sought to find solidarity and community-building with peers.

The pre- and post-course CHANT results highlight that learners were familiar with the impacts of climate change, highly motivated when considering climate-protective behaviors and discussions, and already practicing some climate-protective practices. Importantly, learners reported that despite being highly motivated, they were less likely to practice climate-protective behaviors at work and discuss the health impacts of climate change in professional settings with colleagues, patients, and clients. Identifying the barriers preventing meaningful changes and dialogue is critical to maximize the potential of learners wanting to address the health impacts of climate change at work. Our findings are consistent with other reports utilizing CHANT (13, 18), which found that respondents performed more climate-protecting behaviors at home and lower levels at work. Rangel et al. (18) suggested organizational barriers, less control over practices at work than at home, and the complexity of work in healthcare as impeding proactive behaviors at work. They suggested a focus on barrier removal by the healthcare system to allow more proactive behaviors by healthcare workers.

While learner performance in this pilot course was not formally measured using a specific competency framework, our CHANT findings highlight opportunities for future curricular refinement through more deliberate mapping and competency-based evaluation. For example, use of the CHANT tool provided insight into learners’ baseline awareness, motivation, and behaviors related to climate and health, helping identify both strengths and gaps in climate-informed preparedness among healthcare professionals. These findings can inform alignment with emerging climate and planetary health competencies, with implications not only for this specific course but also for discipline-specific and broader interprofessional competency development (19). Complementary course evaluation data further assessed how well course content, workload, and learning activities aligned with stated objectives, offering additional insight into how climate-health competencies were introduced and reinforced within the curriculum.

Significant changes were not found when comparing the observation of climate-related health impacts like vector borne illnesses and extreme heat illness. This may be due to the course content itself; we note that certain body systems, like the respiratory system, may illustrate clear examples of climate-related health impacts that learners subsequently observe. Since climate affects all body systems, it will be important to innovate in providing examples for those other body systems that are less conspicuously impacted. However, many students in this pilot course were not yet in their clinical rotations and therefore did not have the opportunity to witness the breadth of climate-related health impacts. Therefore, it is pertinent to follow-up with a longitudinal cohort of learners to assess if increases in knowledge and behaviors occur later and following clinical rotations than assessed in the present study. Finally, in future iterations of this course, it will be crucial to increase enrollment of learners who are less familiar with the impacts of climate change to accurately measure the utility of the course materials.

Learners appreciated the interprofessional nature of the course and the in-person opportunities for engagement with each other and with communities. Some learners expressed the desire for more content specific to their individual profession and to understand what other professions’ roles would be in responding to climate-related health scenarios. In retrospect and after reviewing student evaluations, we recognize the benefit of hosting an in-person climate-related case study activity where students could play the part of their chosen profession and engage with students from other professions to discuss the various perspectives and roles they would offer during the case. Therefore, we are hopeful that continued service activities in future course iterations will provide opportunities for students to collaborate and build interprofessional relationships beyond the course. The service opportunities organized by the course will continue requiring that our CoP builds mutually beneficial relationships with community partners to ensure our work is grounded in local community needs.

The final project in the course encouraged students to reflect deeper beyond the content in the modules and find a creative method to inform others about the impact of climate change on health. The submissions focusing on community education, opinion pieces for academic journals, and poems underlies the impact our course had on our learners, moving them beyond a reflection on their own personal growth from this course to sharing their newly gained knowledge with different communities. Although the quality of the submissions was high, the number of submissions was limited to 12, which may be due to the previously highlighted challenges related to time-constraints.

This pilot course faced several limitations. Overall, our learners come from a singular, private institution in the southeastern region of the United States so their learning experiences and perspectives may not represent the experiences of all potential learners. Additionally, as with all scholarship, the course design and related publication are both inherently limited by the positionality of the creators themselves. Epistemology is always informed by one’s background, identities, and lived experiences. Although the instructor team included multiple identities and lenses across multiple facets of diversity (race/ethnicity, gender identity and expression, sexual orientation, age, education, profession and socioeconomic status) and attempted to center historically excluded perspectives in course content, such as those of Black, Indigenous, and People of Color (BIPOC), persons with disabilities, and socioeconomically disadvantaged communities, some viewpoints were likely not included. Additionally, the fact that our course experience was not a credit-bearing course likely limited the engagement of our learners, all of whom were students or faculty in demanding training programs. We also acknowledge that the voluntary nature of this course made these participants more likely to be interested in and motivated towards climate-promoting behaviors. Since information about climate change is ubiquitous in society and media at present, our participants might have learned about climate and health through other means while they were engaged in this course experience, and this could confound our evaluation of what they gained from our course experience alone.

Our CoP competed for and was selected by the Duke Climate Commitment and Duke Learning Innovation to develop a Coursera online course on Climate and Health for Health Professionals. The non-credit bearing Coursera course now allows completely asynchronous learning and is open to learners both inside and outside of Duke University. We are now considering using the Coursera in tandem with some in-person activities for a hybrid credit bearing course at Duke. We are actively discussing ways to increase in-person activities to promote feelings of connectedness with other learners and potentially improve retention, while recognizing that the offered in-person activities were not well attended and there are scheduling limitations of the participating professional programs. As our pilot course modules were based on the globally developed Planetary Health Framework, we are hopeful the adaptation to Coursera will be informative to educators and learners at the international scale, furthering cross-cultural and interprofessional discussions on global climate change and health.

## Data Availability

The original contributions presented in the study are included in the article/Supplementary material, further inquiries can be directed to the corresponding author.

## Supplementary material

SUPPLEMENTARY FIGURE S1 Results summarizing how often learners observe health conditions in patients that are worsened by climate change according to the centers for disease control.

SUPPLEMENTARY FIGURE S2 Results summarizing how often learners perform climate-protective behaviors at home.

SUPPLEMENTARY FIGURE S3 Results summarizing how often learners perform climate-protective behaviors at work.
