# Supplementary figures and images for: A pilot course on climate and health for students and faculty in the health professions
Source: Front Public Health. 2026 Apr 7;14:1714680. doi: 10.3389/fpubh.2026.1714680 (PMC13096033; doi:10.3389/fpubh.2026.1714680)

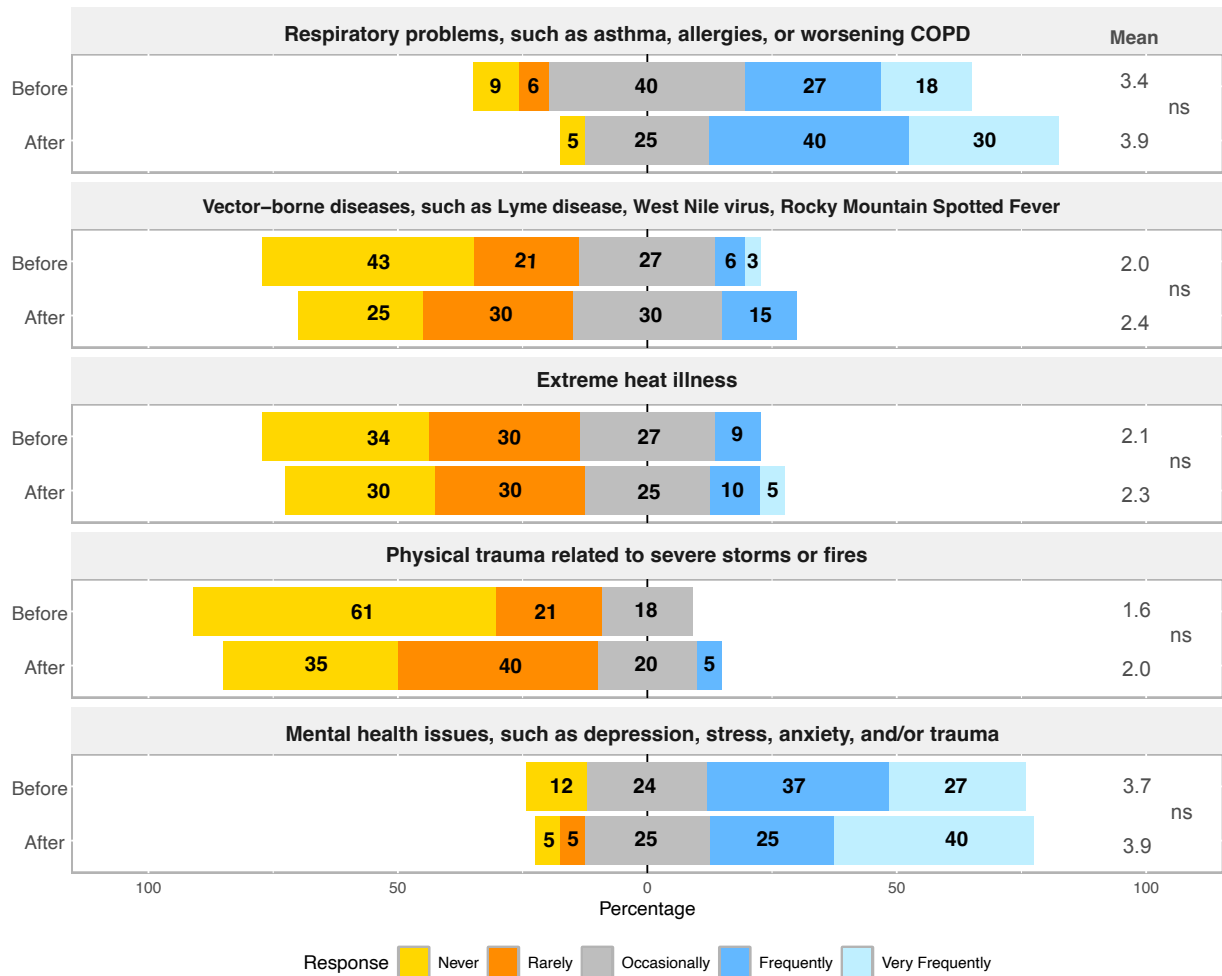

Supplement: SUPPLEMENTARY FIGURE S1 — Results summarizing how often learners observe health conditions in patients that are worsened by climate change according to the centers for disease control. [file Image_1.pdf]

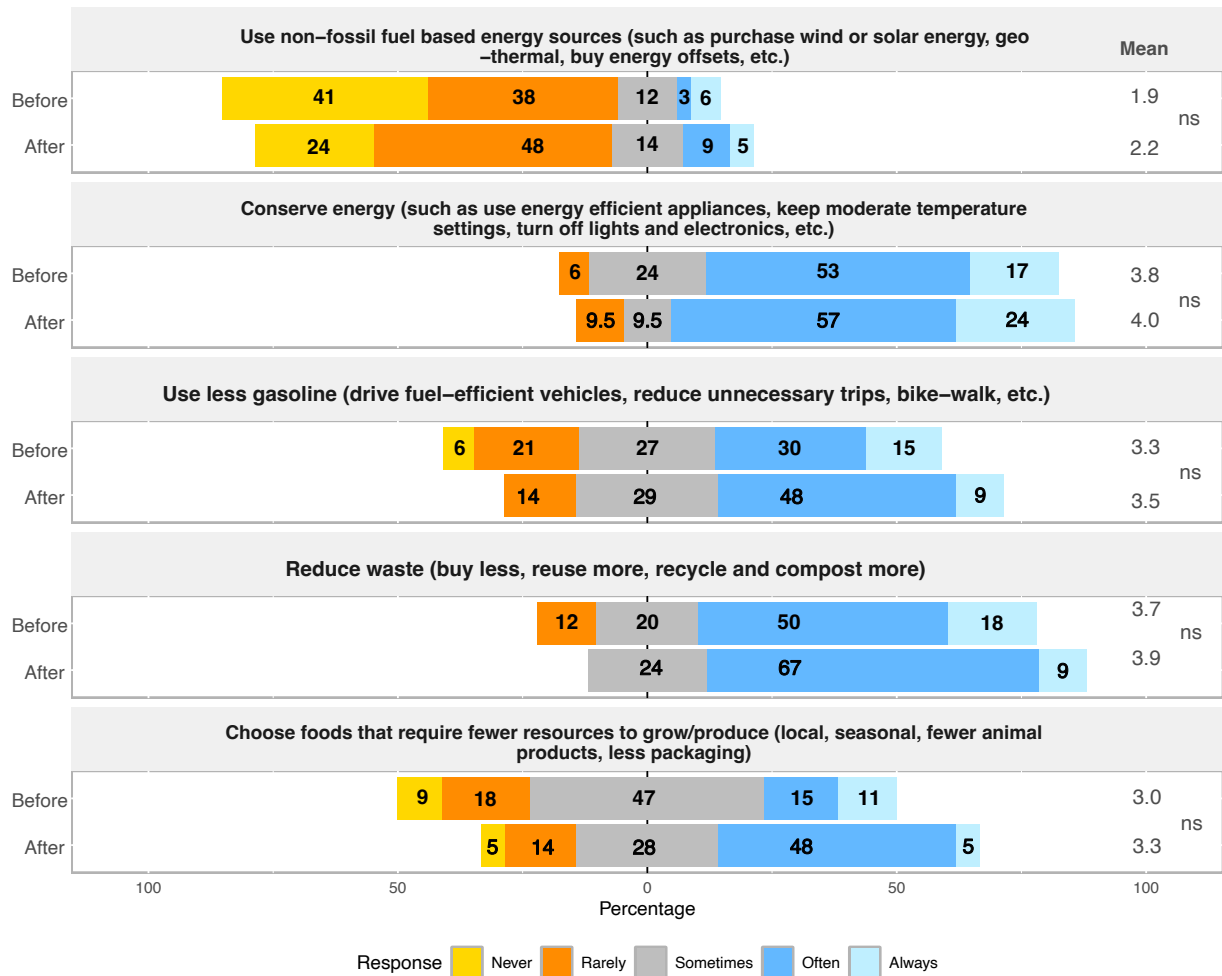

Supplement: SUPPLEMENTARY FIGURE S2 — Results summarizing how often learners perform climate-protective behaviors at home. [file Image_2.pdf]

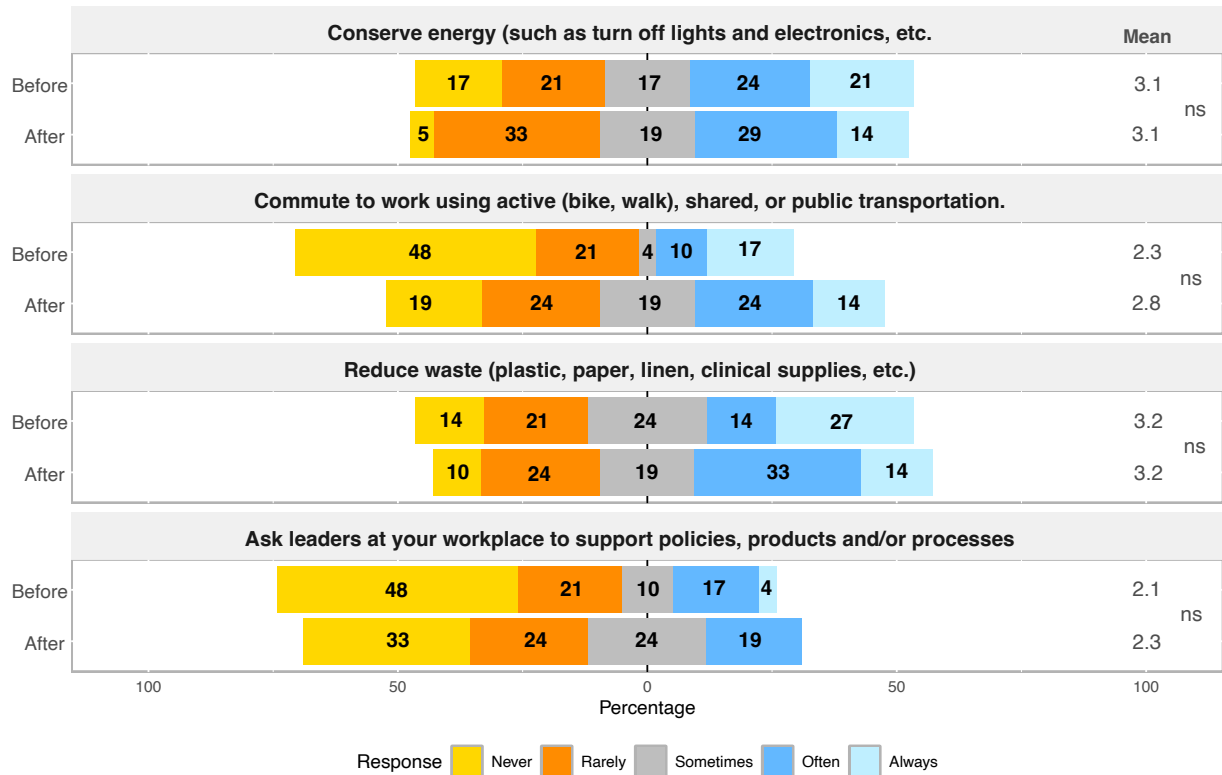

Supplement: SUPPLEMENTARY FIGURE S3 — Results summarizing how often learners perform climate-protective behaviors at work. [file Image_3.pdf]
